# Examining the Efficacy of Control Groups in Achieving Statistical Control: A Critical Look at Randomized Controlled Trials

**DOI:** 10.7759/cureus.70562

**Published:** 2024-09-30

**Authors:** Frederick Strale

**Affiliations:** 1 Biostatistics, The Oxford Center, Brighton, USA

**Keywords:** control group, experimental research, longitudinal study, randomized controlled trial (rct), repeated measures design, statistical control

## Abstract

The randomized controlled trial (RCT) is widely esteemed as the gold standard of experimental research methodologies, purportedly due to its rigorous approach to achieving statistical control. By systematically assigning participants to either a control group or an experimental group through randomization, RCTs claim to isolate the effects of the intervention from confounding variables. This methodological rigor is believed to be instrumental in ensuring that observed outcomes can be attributed with a high degree of confidence to the experimental treatment rather than to extraneous factors. Random assignment in RCTs is believed to mitigate selection bias and enhance generalizability. However, they necessitate a large sample size and are often constrained by ethical considerations.

The repeated measures design represents a sophisticated alternative that provides nuanced statistical control by allowing each participant to serve as their own control. Repeated measures analyses commonly include the paired t-test, Wilcoxen Signed Rank Test, and the Repeated Measures Analysis of Variance (ANOVA). These approaches are particularly advantageous in mitigating the impact of individual variability, an inherent noise source in many research settings. By employing repeated measures, researchers can achieve heightened precision in estimating treatment effects, as each subject's baseline characteristics and responses to experimental conditions are held constant across the various stages of the study.

This nuanced control contrasts with the traditional claim within medical science on the "rigorously controlled" nature of RCTs. While RCTs are celebrated for their methodological robustness and capacity to minimize bias through randomization, their application is not always the most efficient or practical for all research questions. Although significant, the methodological strengths of RCTs may be overshadowed by the inherent limitations of their design, including the inability to "control for" an infinite number of confounding variables, ethical considerations, and the challenge of achieving generalizability across varied real-world contexts.

In contrast, the often-underutilized repeated measures design offers a valuable alternative by harnessing within-subject comparisons to enhance statistical sensitivity. This design is particularly effective when longitudinal data is paramount or focuses on assessing dynamic changes over time as the result of treatment. It is imperative, however, to acknowledge that repeated measures designs have challenges. Potential issues such as carryover effects, order effects, and the complexity of statistical analysis necessitate careful consideration and robust methodological strategies to ensure valid interpretations of the data.

While RCTs remain the gold standard for their claimed methodological rigor and ability to establish causal relationships with high confidence, repeated measures designs offer a complementarily more progressive approach that enhances precision by controlling for individual differences. Both methodologies hold significant merit within the research landscape, and their application should be thoughtfully considered based on the specific research objectives, practical constraints, and the nature of the phenomena under investigation.

## Editorial

At its core, experimental research's primary objective within the scientific domain is to evaluate the efficacy of a particular intervention or treatment rigorously. This process systematically assesses whether the treatment achieves its intended outcomes under controlled conditions. By employing robust methodologies, such as randomized controlled trials (RCTs), researchers can supposedly isolate the effects of the treatment from other confounding variables, thereby ensuring the validity and reliability of the findings. RCTs are considered the gold standard in experiments, advancing our understanding of the treatment’s effectiveness and contributing to the broader scientific knowledge, ultimately informing clinical practices and guiding future research endeavors [1].

The first official RCT to be completed and published was conducted by the United Kingdom’s Medical Research Council in 1947. This trial compared streptomycin and bed rest against bed rest alone for treating tuberculosis. This trial, led by Austin Bradford Hill and Richard Doll, is often cited as one of the first modern RCTs. It used randomization to allocate patients to either receive streptomycin or a placebo, thus establishing a rigorous method for assessing the drug’s efficacy and safety. This study was published in the British Medical Journal (BMJ) on October 30, 1948. You can find the study here: https://www.bmj.com/content/2/4582/769 [2,3].

Repeated measures designs such as the Paired t-test, the Wilcoxon Signed Rank Test, and Repeated Measures Analysis of Variance (ANOVA) merit more attention in experimental data analysis in medical science. These sophisticated methodologies achieve a heightened degree of "statistical control." These techniques are predicated on measuring the same subjects across multiple time points or conditions using the same measurement instrument, allowing the subjects to act as their own controls [3-5]. 

More recognition is needed, as the essence of repeated measures designs lies in their ability to reduce the variability that typically arises from differences between individual subjects. By repeatedly measuring the same subjects, researchers can more precisely attribute observed differences to the interventions or treatments being studied rather than to individual variability that might bias comparisons between control and experimental groups. For instance, in a Paired t-test, differences between paired observations (e.g., pretest and posttest scores) are examined, which inherently controls for individual differences. In parallel, the Wilcoxon Signed Rank Test, a non-parametric alternative, assesses changes in paired observations by ranking the differences between paired measurements [3-5]. 

Repeated Measures Analysis of Variance (ANOVA) extends this concept to more complex experimental designs, where multiple measurements are taken at several time points or under various conditions. This method allows for the analysis of variation within subjects across these multiple levels, thus controlling for between-subject variability and providing a clearer picture of the treatment effects. By comparing the variability within subjects to the variability between subjects, Repeated Measures ANOVA can more precisely isolate the impact of the treatment while accounting for potential threats to internal validity [4]. 

In contrast, using a control group aims to achieve statistical control by matching it with control and experimental groups. This approach typically involves statistical tests such as chi-squares, Fisher's exact tests, and two independent sample t-tests to ensure that groups are equivalent on key demographic variables, ideally resulting in non-significant p-values (p>0.05), which suggest group equivalence. Nonetheless, this method has inherent limitations. Even with rigorous matching on several demographic factors, often up to many variables, there remains a multitude of other variables that may not be controlled for, potentially introducing unaccounted variability that could confound the results [3,6,7]. 

In Propensity Score Matching (PSM), for instance, the number of variables (or covariates) matched can vary widely depending on the study’s design and objectives. Typically, researchers aim to match on a set of critical covariates that are believed to influence both the treatment assignment and the outcome. On average, studies often match five to 20 variables. However, the exact number can be higher or lower based on the complexity of the research and the availability of relevant data [8-10]. What about the infinite number of variables that cannot be matched (controlled for)?

Consequently, while control groups offer a seemingly valuable means of comparison, repeated measures designs provide a more nuanced and robust framework for controlling individual differences and isolating the effects of the treatment or intervention. This approach matches the same subjects across two or more measurements, enhancing the analysis's precision and reducing the risk of confounding variables impacting the findings [3,4]. Given this, the medical research community often extols the virtues of "rigorously controlled studies," RCTs are heralded as the "gold standard" for evidentiary rigor. 

The acclaim bestowed upon RCTs stems from their capacity to supposedly minimize selection bias and confounding variables through randomization, thus enhancing the study's validity. By randomly assigning participants to treatment or control groups, RCTs seemingly ensure that the groups are comparable at baseline, thereby isolating the effect of the treatment from other extraneous influences [1,3,6,7]. 

While RCTs are lauded for their alleged methodological rigor, it is imperative to scrutinize and critically evaluate the extent to which they truly achieve "statistical control" and validity in practical settings. Although a powerful tool for reducing bias, randomization does not eliminate the risk of confounding variables nor addresses all dimensions of variability (noise) affecting study outcomes.

The efficacy of randomization in achieving comparability relies on the assumption that the sample size is sufficiently large and that random assignment is executed flawlessly. In reality, deviations from ideal randomization procedures or inadequate sample sizes can compromise the integrity of the groups, thus impacting the conclusions' validity. 

By mitigating noise, repeated measures designs ostensibly enhance replicability due to reduced random variability. This diminution of variability decreases the probability of encountering a "reproducibility crisis," thereby facilitating the attainment of similar results in subsequent studies. The consistent replication of findings is paramount for affirming the generalizability of research outcomes.

Most assuredly, repeated measures designs significantly address the “reproducibility crisis” by enhancing statistical power and reducing the likelihood of false negatives. They require fewer participants, making studies easier to replicate. By using the same participants across conditions, these designs minimize variability due to individual differences, leading to more reliable and stable results. They also effectively control for confounding variables, reducing noise and isolating the effect of the independent variable. This design’s ability to reduce error variance and increase precision facilitates consistent replication across studies, which is crucial for generalizability. Additionally, collecting data at multiple time points provides a richer dataset, helping to identify patterns and trends, thereby contributing to more comprehensive and reproducible research.

The emphasis on randomization and control groups in RCTs does not prevent the importance of other methodological considerations. For instance, the generalizability of findings from RCTs can be limited if the study sample is not representative of the broader population. The rigorous control claimed in RCTs often involves particular inclusion and exclusion criteria, which can lead to results that are not easily extrapolated to real-world settings. Additionally, adherence to controlled conditions may sometimes obscure the complexities and nuances of treatment effects in everyday clinical practice. 

Conversely, repeated measures designs such as the Paired t-test, Wilcoxon Signed Rank Test, and Repeated Measures ANOVA offer well-grounded alternatives for achieving statistical control. By utilizing the same subjects across multiple time points or conditions, these methodologies inherently control between-subject variability, thus providing a more refined understanding of treatment effects. The repeated measures approach allows researchers to track changes within the same individuals over time, which can be particularly advantageous when studying phenomena with temporal dynamics or individualized responses to interventions [4,5]. 

Nevertheless, it is essential to recognize that repeated measures designs have limitations. For example, the potential for carryover effects on the impact of an initial treatment influences subsequent measurements and poses challenges to the validity of the findings. Additionally, these designs often assume that the effects of interventions are consistent over time, which may not hold in all cases. Factors such as practice effects, fatigue, or other temporal influences can introduce biases that may affect the outcomes [4,5]. In some instances, these methods are complementary and can serve different purposes. Repeated measures alone are not always practical and are often used alongside a control group.

While repeated measures designs control for individual differences by having subjects act as their own controls, they still require meticulous consideration of the assumptions underlying the statistical tests employed. For instance, Repeated Measures ANOVA assumes sphericity, which relates to the homogeneity of variances of the differences between conditions. Violating these assumptions can lead to a higher likelihood of type I and type II errors, necessitating corrective measures or alternative methods [4,5]. 

Additionally, seven threats to internal validity always apply as a limitation in repeated measures designs. History points toward extraneous variables not part of the study or any external events that may affect outcomes. Maturation involves age-related bodily changes and includes age-related physical changes that can occur with time, such as hunger, tiredness, fatigue, wound healing, surgery recovery, and disease progression. Testing relates to the notion that the test may affect the subjects' responses when tested again. These are less of an issue when the tests are routine. Instrumentation refers to any change in measurement ability, including any judge and rater. Statistical regression is the tendency for individuals who score extremely high or low on a measure to score closer to the mean of that variable the next time they are measured on it. Selection refers to the potential bias in selecting study participants. Mortality refers to the differential loss of study participants, drop-out rate, or attrition [11,12]. 

While RCTs are considered the gold standard for their methodological rigor and ability to purportedly control confounding variables through randomization, repeated measures designs provide a consummate approach by controlling for individual differences through within-subject comparisons [4,5]. Both methodologies have their respective strengths and limitations, and the specific research questions, the nature of the intervention, and the practical considerations of the study context should guide their application. A nuanced appreciation of these methodologies and their potential pitfalls is essential for advancing research practices and ensuring that findings are robust and applicable to real-world scenarios. 

A repeated measures design reduces variability by comparing each participant's outcomes across one or different conditions, effectively controlling for individual differences that might otherwise introduce noise. This approach minimizes confounding variables related to subject characteristics, such as age, gender, baseline health status, and an infinite amount of potential confounding variables that would exist within a control group situation because each participant acts as their own control.

By focusing on within-subject comparisons, repeated measures designs may lead to more precise estimates of treatment effects, which may offer better insights into the effectiveness of an intervention. This design can also be more efficient, requiring fewer subjects to achieve the same statistical power compared to traditional RCTs with separate control groups.

Therefore, repeated measures designs often provide a clearer and more accurate picture of the treatment effect by tightly controlling for individual differences. This can potentially enhance the applicability of the results to a broader population, especially in studies where individual variability is a significant factor.

Conclusion

Ultimately, repeated measures designs stand out for their ability to significantly reduce variability by having each participant serve as their own control, inherently accounting for individual differences (e.g., age and gender). This results in greater precision and higher statistical power, often with fewer participants. The ability to gather more detailed, longitudinal data makes repeated measures especially valuable for studies where participant recruitment is difficult or when the goal is to isolate the true effects of the independent variable with minimal noise from extraneous factors. This design being more efficient and effective when controlling for individual differences is critical.

While RCTs rely on randomization to supposedly balance individual differences, claiming to establish clearer cause-and-effect relationships, this process is not always perfect and can require large sample sizes to achieve sufficient power. The increased participant demands and between-group variability make RCTs less efficient, especially in studies with limited resources or smaller sample sizes.

In many cases, repeated measures provide a more powerful, nuanced, and resource-efficient approach than RCTs, particularly when controlling individual variability. Both designs have their place, but repeated measures offer compelling advantages in many research contexts.
